# Substrate-Mediated Laser Ablation under Ambient Conditions for Spatially-Resolved Tissue Proteomics

**DOI:** 10.1038/srep18135

**Published:** 2015-12-17

**Authors:** Benoit Fatou, Maxence Wisztorski, Cristian Focsa, Michel Salzet, Michael Ziskind, Isabelle Fournier

**Affiliations:** 1 Univ. Lille, INSERM, U1192 - Laboratoire Protéomique, Réponse Inflammatoire et Spectrométrie de Masse-PRISM, F-59000 Lille, France; 2 Univ. Lille, CNRS, UMR 8523 - PhLAM - Physique des Lasers Atomes et Molécules, F-59000 Lille, France

## Abstract

Numerous applications of ambient Mass Spectrometry (MS) have been demonstrated over the past decade. They promoted the emergence of various micro-sampling techniques such as Laser Ablation/Droplet Capture (LADC). LADC consists in the ablation of analytes from a surface and their subsequent capture in a solvent droplet which can then be analyzed by MS. LADC is thus generally performed in the UV or IR range, using a wavelength at which analytes or the matrix absorb. In this work, we explore the potential of visible range LADC (532 nm) as a micro-sampling technology for large-scale proteomics analyses. We demonstrate that biomolecule analyses using 532 nm LADC are possible, despite the low absorbance of biomolecules at this wavelength. This is due to the preponderance of an indirect substrate-mediated ablation mechanism at low laser energy which contrasts with the conventional direct ablation driven by sample absorption. Using our custom LADC system and taking advantage of this substrate-mediated ablation mechanism, we were able to perform large-scale proteomic analyses of micro-sampled tissue sections and demonstrated the possible identification of proteins with relevant biological functions. Consequently, the 532 nm LADC technique offers a new tool for biological and clinical applications.

Technological evolution in the field of MS over the past decade has promoted the development of new tools for analyses of crude samples or object surfaces. Accordingly, ambient MS has become a powerful tool for direct analysis of native samples with minimal sample preparation. Consequently, fields of application of ambient MS have widened to human health and environmental studies, as various sensitive objects and organisms such as living tissues or plants can be analyzed using this technique^1^. Most of the ambient MS techniques are either derived from Electrospray Ionization (ESI) or from laser based technologies. The former includes Desorption ElectroSpray Ionization (DESI)^2^,^3^ and Easy Ambient Sonic Spray Ionization (EASSI)^4^, both generating ions by exposing the sample to charged droplets, whereas the latter includes Laser Desorption Ionization (LDI) or Laser Ablation (LA). Several concepts have been explored in the field of laser-based ambient MS techniques. Depending on the technique, both desorption and ionization of molecules are either achieved directly from a pristine sample or from a mixture of the sample embedded within a suitable matrix material. Techniques involving a matrix material are derived from Matrix Assisted Laser Desorption/Ionization (MALDI) and include Atmospheric Pressure MALDI (AP-MALDI)^5^,^6^,^7^. Desorption and ionization can also be decoupled when molecules are first ejected, and then captured and subsequently ionized. The capture is commonly achieved by a spray of charged solvent droplets that are delivered under ESI conditions, allowing for direct analysis of the sampled material by MS. Techniques using this procedure are Laser Desorption Electrospray Ionization (LDESI)^8^,^9^, Matrix-Assisted Laser Desorption Electrospray Ionization (MALDESI)^10^,^11^, Electrospray Laser Desorption Ionization (ELDI)^12^, Laser Electrospray Mass Spectrometry (LEMS)^13^,^14^ or Laser Ablation Electrospray Ionization (LAESI)^15^. Alternatively, ejected molecules can be captured into neutral solvent droplets followed by indirect MS analysis for Laser Ablation/Droplet Capture (LADC)^16^,^17^,^18^,^19^ or be captured by a continuous flow of solvent for direct MS analysis^20^,^21^. These laser-based techniques generally require the use of a wavelength that can efficiently excite the molecules present in the system (matrix or analytes). Accordingly, the wavelengths usually belong to the UV (e.g., at 266 nm^22^ or 337 nm^12^,^20^) or the IR range (e.g., at 2.94 μm^15^,^16^,^17^,^18^,^19^ in coincidence with the O-H stretching vibrational band or at 10.6 μm^8^ close to C-O vibrational bands). In addition, some studies have also tested the use of visible laser light tuned mostly at 532 nm, whereby a continuous wave laser in the transmission mode was used to desorb and inject molecules into the MS using ESI ion source^20^,^21^. However, this was only performed with low absorbing molecules. Some further studies reported the absorption at 532 nm of few proteins present in biological tissues, such as hemoglobin or one subunit of cytochrome C without subsequent MS analysis^23^. Shiea *et al.* evaluated the effects of laser light on the desorption and ionization mechanisms in ELDI-MS^22^ using UV (266 nm), visible (532 nm), and near-IR (1064 nm) lasers with or without matrix. They showed that the presence of a matrix did not improve desorption efficiency and that the mixture ion signal originated from the sample exposed to the near-IR laser. In addition, they suspected the importance of the substrate in the desorption/ionization process because of possible heating of the plate material during irradiation. They confirmed their hypothesis using different sample materials. However, the actual assessment of the role played by the substrate itself is not straightforward because the sample was mixed with a dye that also absorbs at the irradiation wavelength.

Here, we explore the potential of a 532 nm LADC system, with conventional reflection geometry, as a means for tissue micro-sampling and subsequent large-scale biomolecule analysis using substrates of different optical properties. For this purpose, we tested a custom-made LADC system on biomolecule standards of different classes (lipids, peptides, proteins) and on biological tissues. After optimization of the LADC system, we were able to demonstrate the existence of a Substrate-Mediated Laser Ablation (SMLA) mechanism that makes possible the analyses of intact biomolecules, analyses performed with equal efficiency whatever are the absorption properties of the sampled system. Finally, we conducted a Shotgun MS analysis of the material collected from histological regions of rat brain tissue sections. We were not only able to identify the proteins in the tissue sections, but also to get their relative quantification under label-free conditions. Accordingly, we were able to determine the efficiency of our system for spatially resolved micro-proteomics with relative quantification.

## Results

### Optimization of Laser Ablation and Droplet Capture on standards

**Figure 1:**
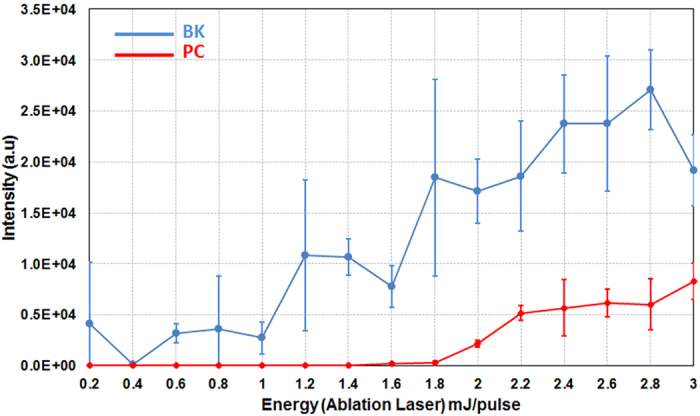
Evolution of the signal intensity as recorded in MALDI MS by analyzing the capture droplet after LADC experiment with the ablation laser energy/pulse for liquid PC (in red) and liquid BK (in blue). Signal intensity corresponds to the averaged intensity of [M+H]^+^ signals taken over the different measurements.

**Figure 2:**
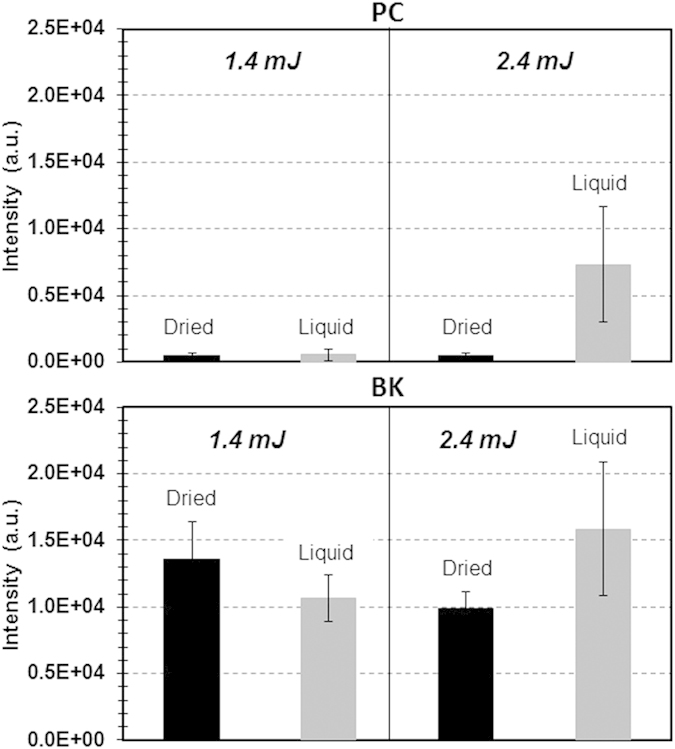
Comparison of the signal intensity as recorded in MALDI MS by analyzing the capture droplet after LADC experiment for PC and BK standards at 1.4 and 2.4 mJ/pulse ablation laser energy for dried (black) and liquid (grey) preparation deposits. Signal intensity corresponds to the averaged intensity of [M+H]^+^ signals taken over the different measurements.

Optimization of the 532 nm LADC technique was carried out on three different standards: Bradykinin peptide (BK), Phosphatidyl Choline lipid (PC) and Lysozyme C protein (LYZ). We first studied and optimized the laser energy of the LADC system. Figure 1 shows MALDI MS signal intensity evolution of BK and PC versus ablation laser energy. The standards were deposited in their liquid form on a stainless steel slide. Similar trends were observed regarding signal evolution of both the peptide and the lipid but with different detection thresholds (~1.0 mJ/pulse for BK and ~2 mJ/pulse for PC). The maximum yield is reached at ~2.4 mJ/pulse for both BK and PC, although the signal intensity is always lower for PC. Beyond the energy of 2.4 mJ/pulse, the respective intensities of BK and PC are approximately constant and both curves begin to plateau. A similar trend is also observed for the LYZ (data not shown). Signal intensities for both liquid and solid BK and PC samples deposited on stainless steel substrates were then compared in Fig. 2. While only low intensity signals were observed for solid PC samples, signals of comparable intensity were noted for solid and liquid BK. This discrepancy can be interpreted as a difference in the behavior of liquid PC and BK samples as they dry out. Because of their physical and chemical properties, lipids suspended in MeOH (solvent) tend to spread out to the periphery of the droplet where they form micro-crystals. This greatly reduces the probability of interaction with the laser that targets the center of the droplet. On the other hand, BK forms a polycrystalline film with more evenly distributed micro-crystals, which facilitates interaction with the laser.

**Figure 3:**
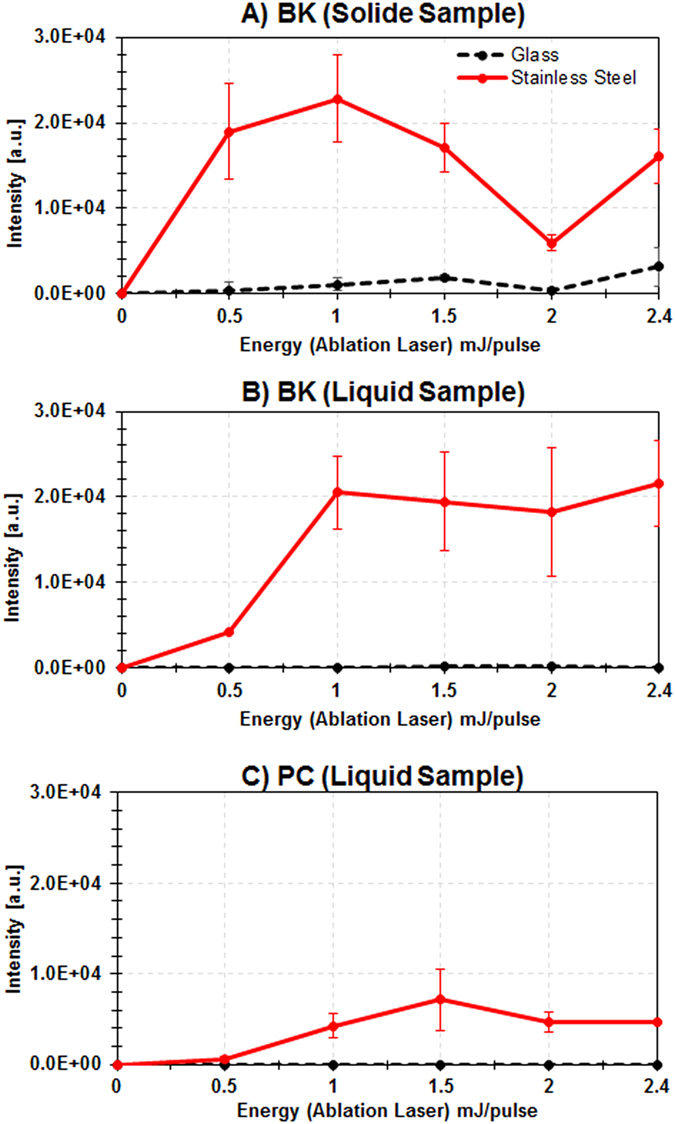
Evolution of the signal intensity as recorded in MALDI MS by analyzing the capture droplet after LADC experiment with the ablation laser energy/pulse from glass slide (black) versus stainless steel (red) substrate for (A) BK solid sample, (B) BK liquid sample and (C) PC liquid sample. Signal intensity corresponds to the averaged intensity of [M+H]^+^ signals taken over the different measurements.

**Figure 4:**
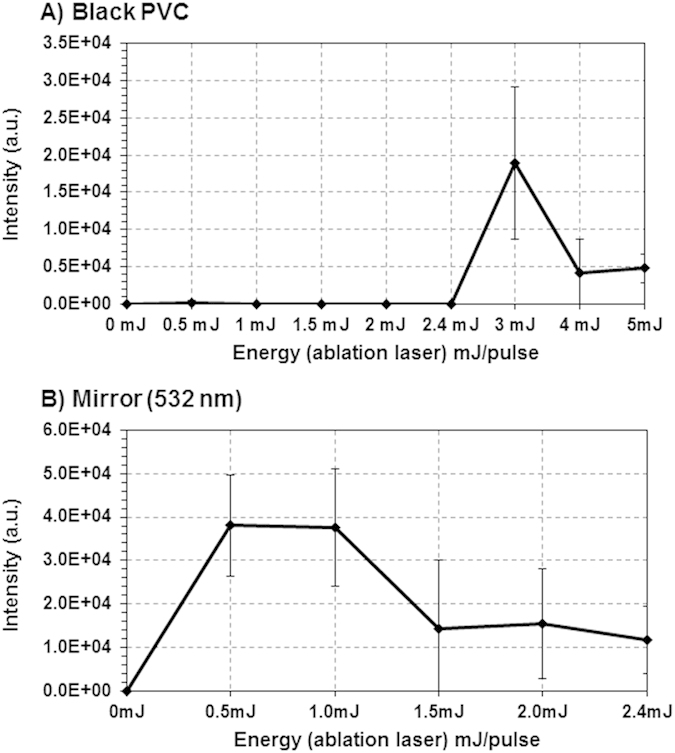
Evolution of the signal intensity as recorded in MALDI MS by analyzing the capture droplet after LADC experiment with the LADC laser energy/pulse for BK solid sample from (A) Black PVC tape and (B) high-reflectivity mirror at 532 nm. Signal intensity corresponds to the averaged intensity of [M+H]^+^ signals taken over the different measurements.

Interestingly, 532 nm-LADC MALDI MS intensity signals recorded from low absorbance (~0.2 cm^−1^) samples exhibit similar intensities than signals recorded from high absorbance (10^3^–10^4^ cm^−1^) samples using a 2.94 μm-LADC system^16^ with a similar fluence. This strongly suggests that the ablation mechanism resulting in the observed intensity signals for low absorbance samples do not originate from a direct interaction between the sample and the laser. Consequently, new experiments were performed to compare signal evolution of a substrate known to be transparent at 532 nm (silica glass) to that obtained previously from stainless steel. These experiments clearly evidenced an effect of the sample plate since little or no signal was observed from the transparent material whatever the sample studied: solid BK (Fig. 3A), liquid BK (Fig. 3B) and liquid PC (Fig. 3C). The origin of this matrix-free substrate-mediated effect can obviously be found in the optical properties of the substrate. For instance, we determined that the reflectivity of stainless steel is about 35%, the remaining 65% of incident light being absorbed or scattered by the substrate. Thus, in order to clarify the contributions of the absorbance and reflectance of the substrate on the subsequent ablation mechanism, we carried out further laser energy studies on pure BK samples deposited on substrates with opposite optical properties (black PVC tape versus high reflectivity mirror). Almost no signal was detected using the black PVC substrate in the range of laser energy previously studied (Fig. 4A). However, the appearance of signal at higher energy (around 3 mJ/pulse) was noted. In contrast, the use as a substrate of a laser mirror coated to enhance its reflectivity at 532 nm led to the detection of the BK signal that exhibits a similar evolution trend as was obtained from BK deposited on stainless steel in the same laser energy range (Fig. 4B). This result tends to exclude absorption mechanisms as being part of the observed ablation process. Consequently, heating processes such as those found in Laser-Induced Thermal Desorption (LITD)^24^, explosive boiling^25^,^26^,^27^ or the emergence of pressure waves (e.g. Laser-Induced Acoustic Desorption (LIAD)^28^,^29^,^30^,^31^) are not expected to take part in the observed ablation mechanism. On the other hand, the importance of reflection in the observed substrate-mediated effect seems rather to favor a multiphotonic mechanism by simultaneous interaction between the molecules in contact with the substrate and the incident and reflective laser beams during the ns laser irradiation. The molecules would then show 2 times higher energy at the most, which could lead to a two-photon absorption process. Since the reached wavelength, 266 nm, is generally characterized by a higher optical absorption, the molecules are excited much more effectively, leading to the excitation of the whole volume and finally to the ablation of neutral material from the sample. The emergence of signal at around 3 mJ/pulse during irradiation of BK deposited on PVC, with an energy that is about twice higher than that required to observe any signal when stainless steel or highly reflective mirrors are used as substrates (see Fig. 4A), could support this assumption. It must be noted that the use of 3 mJ pulses ablates the PVC support but also damages the coating of the mirror, probably via collisions of highly excited molecules impinging on the support. For these reasons, the use of stainless steel with pulse energy inferior to 3 mJ/pulse seems to be the best compromise for our applications.

**Figure 5:**
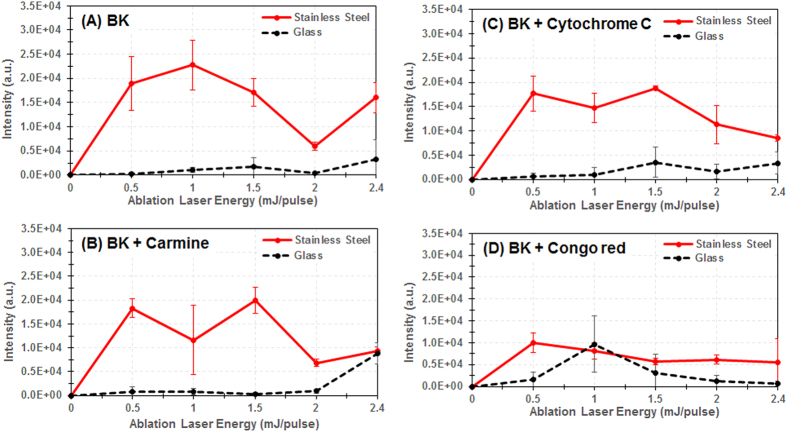
Evolution of the signal intensity as recorded in MALDI MS by analyzing the capture droplet after LADC experiment with the LADC laser energy/pulse from glass slide (black) versus stainless steel (red) substrates for solid BK (A), solid BK mixed with carmine (B), solid BK mixed with cytochrome C (C), and solid BK mixed with Congo red (D). Signal intensity corresponds to the averaged intensity of [M+H]^+^ signals taken over the different measurements.

In order to compare the indirect substrate-mediated ablation to the conventional direct ablation mechanism, we repeated the experiments by adding dyes with different absorption coefficients at 532 nm. In this context, we could expect dye addition to shift the laser energy distribution to lower energies and/or improve the sensitivity. Figure 5 shows the evolution of the MALDI MS signal intensity with increasing ablation laser energy for dried BK alone (optical absorption coefficient at 532 nm ~0.2 cm^−1^) (Fig. 5A) versus BK-carmine (α = 1 cm^−1^, corresponding to an increase of absorbance by a factor ~5 compared to pure dried BK sample) (Fig. 5B), BK-cytochrome C, (2.4 cm^−1^, ×15) (Fig. 5C), and BK-Congo red (28 cm^−1^, ×140) (Fig. 5D) all deposited either on stainless steel or on silica glass. This study demonstrates a different behavior than that expected. The signal acquired from a silica glass substrate is negligibly influenced by the addition of carmine or cytochrome C. Only the Congo red gives rise to a relatively higher signal around 1 mJ/pulse, but this signal is still about half than that obtained from the pure sample deposited on a stainless steel substrate (Fig. 5D). A progressive decrease in signal intensity with increasing dye absorption coefficient is generally observed from mixed samples deposited on stainless steel substrates. In fact, we observe a decrease in signal intensity by at least 50% in the whole tested energy range when comparing irradiation of the BK-Congo red mixture to the pure BK sample, both deposited on stainless steel. This behavior could be explained in the framework of the substrate-mediated effect, as evidenced by the measurement of the fluence at the interface sample/substrate that shows a decrease of about 25% for the BK-Congo red mixture. The decrease is logically less marked for BK-carmine (Fig. 5B) and cytochrome C (Fig. 5C) since only ~5% and ~10% of the energy was absorbed by the sample.

In summary, our experiments highlight the existence of an indirect ablation process, referred to as Substrate-Mediated Laser Ablation (SMLA). This mechanism results from the interaction between the laser and the substrate. The latter can assist or even replace direct ablation mechanisms when the absorbance of the sample is too low. At 532 nm, where almost none of the biological molecule shows significant absorption bands, the substrate advantageously replaces direct ablation process and generates high signal intensities at relatively low laser energy thresholds. Worthy of note, the efficiency of this indirect substrate-mediated ablation mechanism is even higher than that of the direct ablation effect, as demonstrated by the addition of dyes showing absorption at 532 nm. Finally, SMLA is largely independent of the nature of the sample (liquid, dried) and of the standard (lipid, protein, or peptide).

Some other optimizations were performed on the LADC system. One is the study of how peak intensities in MALDI MS spectra vary with the distance between the sample and solvent droplets. This optimization work was performed using solid BK samples deposited on stainless steel slides (Figure S-1). Comparison of BK signals with respect to the distance shows unsurprisingly a highest ablation yield when the droplet was very close to the sample. Beyond 2 mm, the signal rapidly decreased and became nearly undetectable at 4 mm. This observation is consistent with the length of the ablated plume, limited to a few mm due to its interaction with the atmosphere^23^,^32^,^33^. Another explanation to the observed progressive signal extinction could be that the radial distribution of the plume is such that beyond 2 mm there is too much divergence for all the ejected materials to be captured in the droplet^34^. To confirm or infirm the latter hypothesis, we performed LADC experiments at the same distances but using different volumes of droplets. We found that the signals observed in the MALDI spectra are very similar (data not shown) regardless of the tested droplet volume (2 μL or 0.5 μL). This demonstrates that the plume radial distribution is not the most critical parameter. Moreover, we studied the influence of the number of laser shots on the captured material. As expected, an increase of signal intensity with the number of laser shots was observed for both BK and PC (Figure S-2). These experiments were performed at the laser energy giving the maximum signal intensity for each molecule, i.e., 2.4 mJ/pulse for PC (liquid) and 1.4 mJ/pulse for BK (solid). For PC, a 4-fold increase in signal intensity was observed between 10 and 100 laser shots, whereas only a factor of 1.5 was measured for BK in the same conditions. The signal intensity does not increase linearly with the number of laser shots. This indicates that most of the material is ablated and captured before the number of shots reaches 100.

### Optimization of the LADC system on biological tissue sections

**Figure 6:**
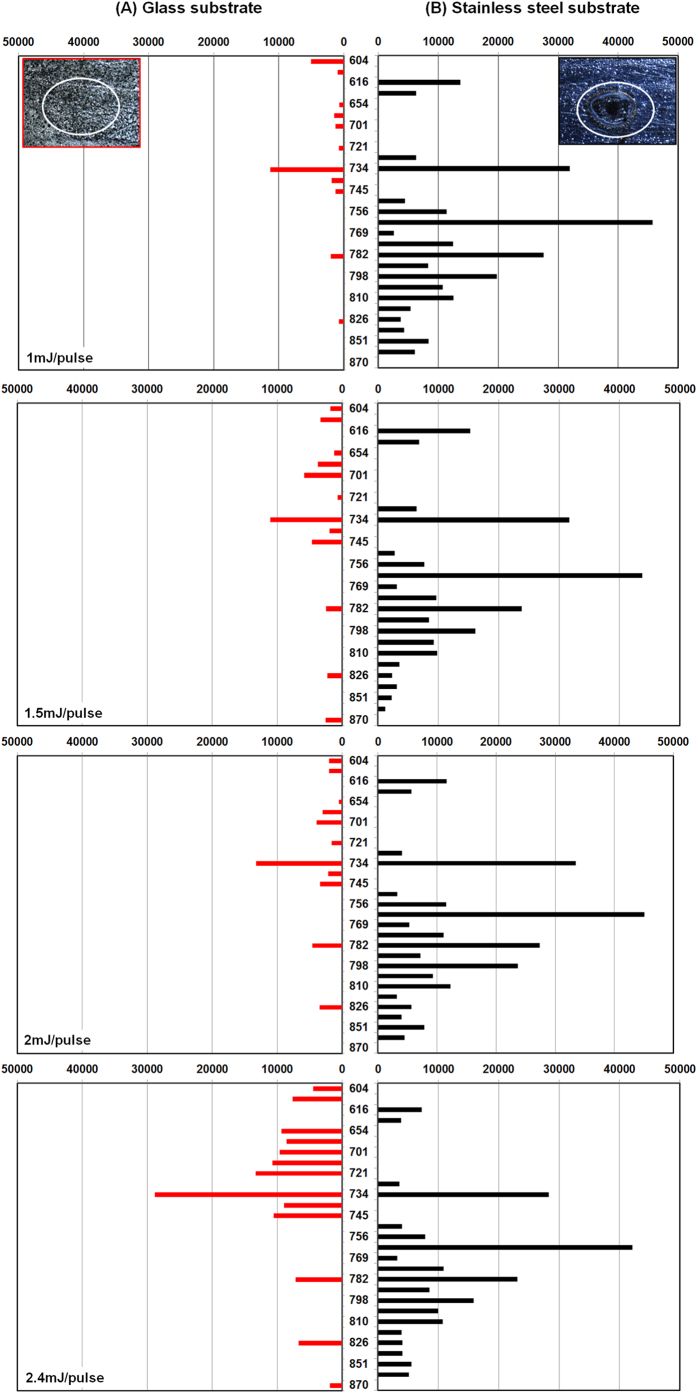
Evolution of the signal profiles (for most significant signals) from (A) glass substrate and (B) stainless steel substrate on a rat brain tissue section (60 μm thickness) for 1.0, 1.5, 2.0 and 2.4 mJ/pulse ablation laser energy. Inserts show a microscopic observation of the irradiated tissue area from glass substrate (left) and stainless steel substrate (right). Each bar represents the sum of the signal intensity over the 9 experiments.

In general, tissues do not show important absorption at 532 nm, except for certain molecules such as melanin and blood that exhibit slightly higher absorption^35^. The overall capacity of tissues to absorb light is therefore dependent on both their melanin and blood content but should remain low in general. We thus expect the direct ablation mechanism, whereby the laser directly interacts with the tissue sample, to make only a small contribution to the whole ablation process, particularly at the laser energy used. Furthermore, we expect the indirect substrate-mediated laser ablation mechanism that we observed during examination of our standards to compensate for the direct mechanism. Figure S-3 presents MALDI MS spectra recorded after LADC experiments operated at different laser ablation energies on 60 μm thick rat brain tissue sections. Various signals that might correspond to metabolites and lipids emerge in the LADC analysis, most of them lying in the 400–1000 m/z range (below m/z 400, signals were attributed to the matrix). Few signals of lower intensity were also observed in the 1000–5000 m/z range (data not shown), which might be attributed to larger lipids and/or few endogenous peptides. No signals are observed above m/z 5000. The selective observation of metabolites and lipids can be explained by their large abundances within the analyzed tissues and by the manifestation of ion suppression effects during MALDI MS analyses of such complex mixtures. In fact, microscopic observations of the irradiated spot showed that all the tissue is ablated after 100 laser shots. This means that tissue pieces might also be ablated under our conditions. Confirmation was obtained when some droplets emanating from the LADC were collected and examined under a microscope. The collected droplets showed the presence of small pieces of tissue material and inclusions of lipid droplets. However, it is well known that for MS of complex mixtures, important ion suppression effects are observed, promoting certain classes of molecules among others. With respect to the evolution of signals with the laser ablation energy, we observed a global increase in the number and intensity of the detected signals between 0.6 and 2.4 mJ/pulse especially in the 700–900 m/z range. On the other hand, certain signals, especially those under m/z 700, were not observed anymore at the highest energy (3 mJ/pulse). Interestingly, evolution with laser ablation energy of the signal acquired from the tissue section relates to what was observed for PC lipid standards, where signal maxima emerged in both samples at around 2.4 mJ/pulse. In contrast, metabolites give the most intense signals at lower laser energy (<1.4 mJ/pulse). The detected m/z were then identified by lipid extraction from a consecutive rat brain tissue section and further analysis using ESI-MS & MS/MS on a FTMS instrument. PC lipid species are identified by their MS/MS ion signature at m/z 184 and are mostly present in the tissue section. This is not surprising since PC belongs to the most abundant class of lipids in biological tissues. Since 532 nm LADC gave interesting results from tissues, we were interested in the fundamental aspects and mechanisms occurring during analyses. We hence repeated the former experiment in the difference that tissue sections were deposited on different substrates (silica glass, stainless steel). Figure 6 shows the evolution with the nature of the substrate of the intensity of the most significant signals obtained at different LADC laser energies. The most striking fact is that about two-times more signals were observed using the stainless steel substrate compared to the silica glass, along with a 3 to 10-fold increase in signal intensity. This again demonstrates the importance of substrate-mediated effects in our LADC system. Microscopic observations of tissue sections performed after LADC experiments further confirmed the substrate-mediated mechanism (Fig. 6): while tissues deposited on silica glass do not show visible damages, tissues deposited on stainless steel have been totally ablated from the irradiated area. More, several interesting considerations can be made from the observed molecular profiles: i) most of the peaks observed from silica glass are exclusives and not observed from stainless steel and vice versa, and ii) peaks observed from silica glass are also of lower m/z in general. This complementarity in the molecular profiles demonstrates again the coexistence of different ablation mechanisms. In the case of tissues, signals from silica glass highlight the contribution of a direct ablation mechanism due to natural absorption of tissues at 532 nm. Brain tissues are composed for a large part of lipid species and the absorption coefficient of fat mass itself is about 1000 cm^−1^ at 532 nm^36^, the latter clearly not being the optimal absorption wavelength. For example, absorption is greater than 10^4^ cm^−1^ at 900 nm^35^. Other molecules such as hemoglobin or cytochrome C similarly exhibit low absorption properties at 532 nm. Since the absorption coefficient of the tissue analyzed in our experiment is not precisely known, we measured the laser light transmitted through the tissue section deposited on a glass slide. This experiment shows that only 15–20% of the laser light is absorbed and that the remaining 75–80% of the fluence reaches the substrate. The tissue’s natural absorption is thus even lower than that of the BK-Congo red system previously studied, explaining the contribution of the SMLA mechanism despite a tissue thickness of 60 μm. We further studied the evolution at different laser energies of signals acquired from thicker tissue sections deposited on stainless steel substrates. Using manually sectioned 1 mm thick tissues (Figure S-4) we observed a drastic decrease in the signal intensity (more than 10-fold), which is the consequence of the low amount of laser energy reaching the substrate. This demonstrates again the primordial effect of the SMLA mechanism in 532 nm LADC experiments and its preponderance over direct ablation at low laser energies.

### Application of LADC for spatially-resolved proteomics

**Figure 7:**
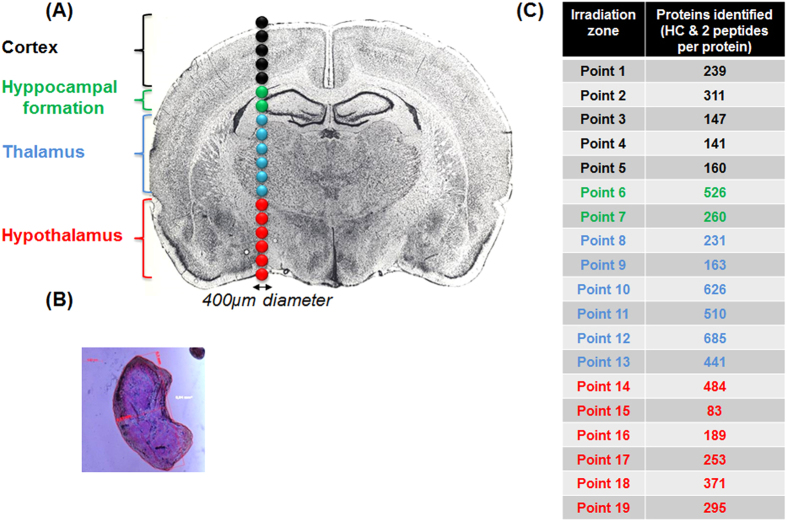
(**A**) picture of the studied rat brain tissue section (60 μm thickness) for Shotgun proteomics experiments showing the 19 irradiated points performed in a row and the corresponding histological regions. (**B**) Microscopic observation of a capture droplet showing the presence of tissue pieces. (**C**) Number of identified proteins for each of the 19 irradiation points.

**Figure 8:**
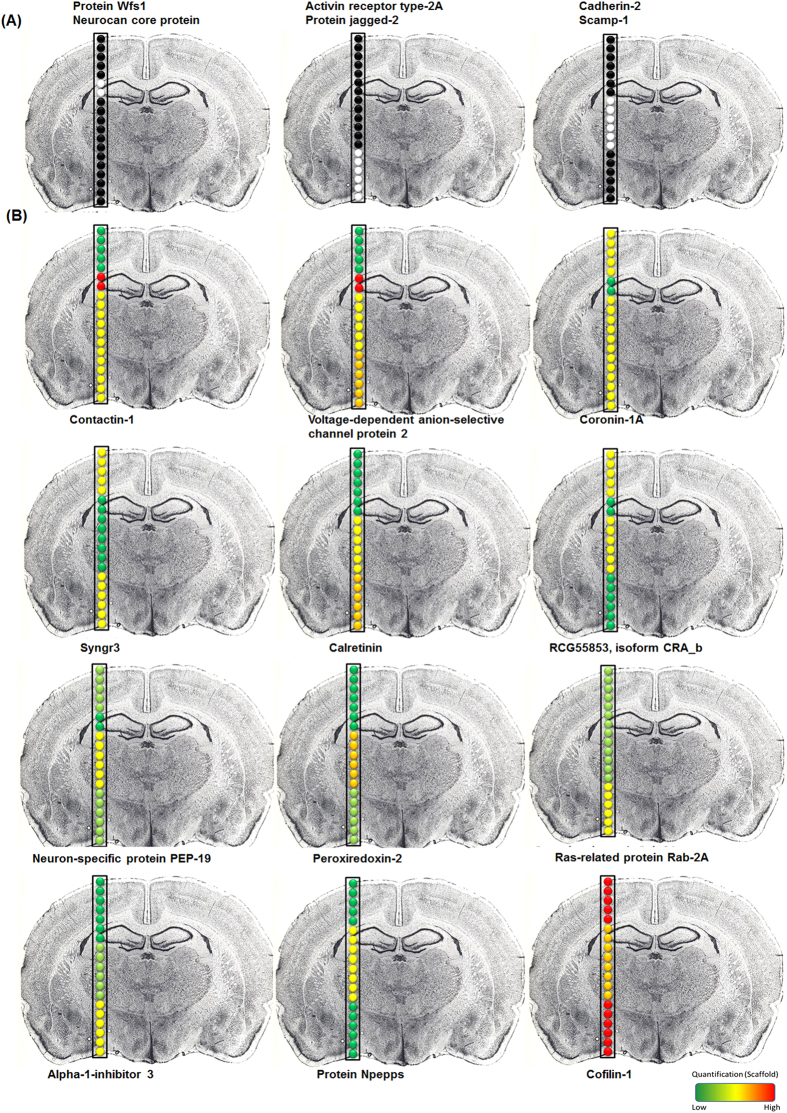
Evolution of the relative quantification for selected proteins identified in Shotgun analysis from the 19 distinct consecutive irradiation points performed in a row on a rat brain tissue section (60 μm thickness). (**A**) Presence (white)/absence (black) and (**B**) relative quantification of the proteins in the different area of the tissue section.

Possible protein identification from very small and specific locations of a tissue is critical for biological and clinical applications to compare cell phenotypes and better understand physiological mechanisms. Different methods are used for this purpose such as Laser Capture Microdissection (LCM)^37^, micro-extraction^38^,^39^ and micro-excision^40^ although there is a real challenge for developing other strategies to be able to reduce the size of the sampled area. Since our LADC droplets contain tissue pieces (Fig. 7), tissue material should be enough for protein analysis using a dedicated proteomics strategy. Shotgun analysis of the proteins contained in the ablated tissues present in the LADC droplets was then performed. Proteins were enzymatically digested and the subsequent tryptic peptides were analyzed by nanoLC-HR MS & MS/MS. The LADC technique was applied on 4 histological regions of a rat brain tissue section (Fig. 7), corresponding respectively to the cortex (region 1), hippocampus (region 2) thalamus (region 3) and hypothalamus (region 4). A line of 19 consecutive irradiations, with a surface of 350 × 400 μm, crossing these 4 regions was performed, and the 19 LADC droplets collected out of these irradiations were processed for Shotgun proteomics. An average of 400 proteins per capture was identified with high confidence (FDR 0.1% and 2 peptides per protein). Plotting the identified proteins with respect to their cellular localization (Figure S-5) showed that they are largely distributed proteins in the cytoplasm, the intracellular organelles and different membranes. These results indicate that proteomics from the LADC technique gives access not only to proteins distributed in various compartments of the cells, but also to some membrane proteins. These identified proteins can be grouped into two categories. The first group (Figure S-6A) represents proteins common to the 4 regions (R1-R4) whereas the second group (Figure S-6B) corresponds to the proteins specific to one region. Figure 8A displays the distribution of 6 proteins specific to one region. For example, the proteins Wfs1 and Neurocan core protein (Fig. 8A left panel) were only identified in the hippocampal formation (region 2), Activin receptor type-2A and protein jagged-2 were detected in the hypothalamus (region 3) whereas Cadherin-2 and Scamp-1 were specifically identified in the thalamus (region 4) (Fig. 8). Common proteins to the 4 regions demonstrate a high degree of interconnectivity altogether (Figure S-6A). Regulation with respect to the spatial distribution of these common proteins was then studied using a label-free quantification method (Table 1, Supplementary Information 2). Quantification values were plotted against a color scale as exemplified in Figure 8 for 15 selected proteins. Contactin-1 and Voltage-dependent anion-selective channel protein 2 were highly present in the hippocampus (region 2) compared to the cortex (region 1). In contrast, Syngr3 is more present in the cortex (region 1) and hypothalamus (region 4) regions whereas the Neuron-specific protein PEP-19 is more specifically found in the thalamus (region 3). The proteins Glutamate receptor (Gria2), Arl15, wolframin protein (Wfs1) and Ncan, identified in the hippocampus region, are known to be specific to that region. Gria2 belongs to a family of glutamate receptors and is specifically expressed in Dental gyrus (DG) and CA3^41^. Whereas WFS1 is predominantly produced in selected neurons in the hippocampus CA1^42^. NCAN encodes neurocan, which is known to modulate neuronal adhesion and neurite growth, is localized within cortical and hippocampal areas^43^. These areas are involved in cognition and emotion regulation. All these proteins have been found to be over-expressed in temporal lobe epilepsy^44^. In the thalamus, 58 specific proteins were identified such as the proteins of the ankyrin family, axonal guidance, cell communication, development, metabolism and cell adhesion^45^. The thalamus plays major roles in the central nervous system as it is a relay center for organizing information, such as auditory and visual senses from diverse brain regions and their re-distribution to the cerebral cortex. Several brain diseases including schizophrenia, Parkinson’s disease, epilepsy, and bipolar disorder have been associated with thalamus disorders^46^. Comparison of the proteins identified in our experiments with previous proteomic studies on MK-801-treated rats^46^ using 2D gels or Shotgun based proteomic approaches^47^ demonstrates that the previously identified proteins are found from the LADC sampled tissues (Table 2, Supplementary Information 3). But interestingly the proteins highly specific to the thalamus (region 3) were not previously reported from other proteomic strategies. Other proteins of particular interest include Ctnna2, Cdh2, Insrr, Gsk3a, Olfml2a and Robo1 which are involved in neurites outgrowth as well as brain pathologies. Robo1, Cytoplasmic FMR1 interacting protein 1 (CYFIP) were found to be involved in the outgrowth during development and regenerative sprouting^48^. CYFIP1 is known to be involved in stabilization of mature dendritic spines during neuronal outgrowth^49^. The 5 proteins specifically identified in the hypothalamus (region 4) adam6, acvr2a, Atp8a, jag2 and wap were also revealed to play central roles in pathologies. Plasticity-related gene 1 proteins (PRG-1) are three proteins involved in neurogenesis. Robo1 acts as molecular guidance cue in cellular migration, including axonal navigation, and plays a major role for establishing correct connections^49^,^50^. PRG-1 is known to facilitate axonal guidance and the Whey acidic protein Precursor (WAP) is essential for anosmin-1 biological function in brain^51^. The Protein jagged-2 Fragment (Jagged2) is a putative Notch ligand involved in the mediation of Notch signaling. This protein exerts a role in neurogenesis in the peripheral nervous system^52^. The Atp8a1 protein (aminophospholipid transporter (APLT), class I, type 8A, member 1), Acvr2a (Activin receptor type-2A) and Adam6 play an important role in learning, brain anxiety disorder and nervous system development, plasticity and repair^53^, respectively. Altogether, all data confirm that LADC sampling followed by Shotgun proteomics allows to retrieve particular proteins from specific brain regions which can be correlated with the physiopathological state of the rat. Most of the proteins detected are clearly novel in term of identification based on proteomic technology.

## Discussion

In summary, we have demonstrated that the Laser Ablation and Droplet Capture (LADC) system at 532 nm followed by MS is a powerful technique for tissue micro-sampling towards the analysis of large scale biomolecules such as Shotgun proteomics. Interestingly, we have identified an ablation mechanism mediated by the substrate which is preponderant over conventional laser ablation mechanisms by direct absorption for low-absorbing samples studied in the low laser energy range. This SMLA mechanism is related to the optical property of the substrate and increases with the reflectivity of substrates. Ablation processes involving substrate effects were previously described in the literature. However, these mechanisms are different from the SMLA mechanism presented here since in the former case the substrate generally absorbs part of the light, which results in heating^24^, explosive boiling^25^,^26^,^27^ or in the formation of pressure waves^28^,^29^,^30^,^31^, or then refers to confinement effects such as for nanostructures or nanomaterials^54^,^55^. However, these processes were shown not to occur here. Consequently, the SMLA has many advantages for biological applications since most biomolecules show no or weak absorption at 532 nm. Since the SMLA is driven by the substrate and independent of the analytes, LA systems involving this mechanism are able to analyze all biomolecules. In addition, because the laser energy used for laser ablation is low, biomolecules are prevented from fragmentations and the sample plate does not suffer from significant damage. Applied on tissue sections, SMLA shows efficient ablation and collection of small tissue pieces within the droplet. While the SMLA efficiency diminishes with tissue thickness, tissue sections up to 60 μm thick can be used. Proteomics performed from a single (2 μL) collected droplet on a rat brain tissue yielded an average identification of 400 proteins from an irradiated surface of ≈350 × 400 μm. Systematic LADC of different morphological regions of the rat brain tissue allows observation of regional regulation of these proteins under label-free quantification conditions which is perfectly in line with the biological knowledge. Interestingly, many of the identified proteins have been previously shown to have implication in neurological disorders or neurodegenerative diseases and a few have never been found before. Thus, 532 nm LADC using the SMLA mechanism can provide a powerful tool for micro-sampling of biological material under ambient condition. It also has high potential for translational clinical applications. Indeed, since the ablation process is independent of the analyte absorption it is possible to provide a constant sampling method only mediated by the substrate. The system is easily compatible with the use of thin tissue section materials such as generally available from the hospitals (patient biopsies). One of the other advantages is that the micro-sampling area can still be decreased by decreasing the laser beam diameter. In preliminary results, we were able to achieve 150 μm micro-sampling area which make this setup advantageous compared to liquid microjunction extraction systems^56^,^57^ which are limited by the size of the micro-capillary used. Finally, since proteomics processing is achieved from the captured droplets it will be usable from both frozen preserved biopsies for prospective studies or from Formalin Fixed and Paraffin Embedded (FFPE) tissues for retrospective analysis. This system will thus provide a novel tool to sample regions of different phenotypes from pathological samples in order to access new potential diagnosis, prognosis markers, or even knowledge on the physio-pathological mechanisms.

## Methods

### Chemicals

All chemicals were of the highest obtainable purity. Water, formic acid (FA), trifluoroacetic acid (TFA), acetonitrile (ACN), and methanol (MeOH) were purchased from Biosolve B.V. (Valkenswaard, the Netherlands), alpha-cyano-4-hydroxycinnamic acid (HCCA), 2.5-dihydroxybenzoïc (2,5-DHB), sinapinic acid (SA), lysozyme C and cytochrome C, ammonium bicarbonate (AB), dithiothreitol (DTT) and iodoacetamide (IAA) from Sigma-Aldrich (Saint-Quentin Fallavier, France), bradykinin (BK) from PolyPeptide Group (Strasbourg, France), Phosphatidyl Choline 15:0/15:0 lipid species (PC) from Avanti Polar Lipid (Alabaster, Alabama, USA), carmine from George T. GURR LTD (London, England) and Congo red from Microcolor (Boulogne, Seine, France). Trypsin (sequencing grade modified trypsin, porcine) was purchased from Promega (Charbonnieres, France).

### Laser Ablation and Droplet Capture (LADC) setup

**Figure 9:**
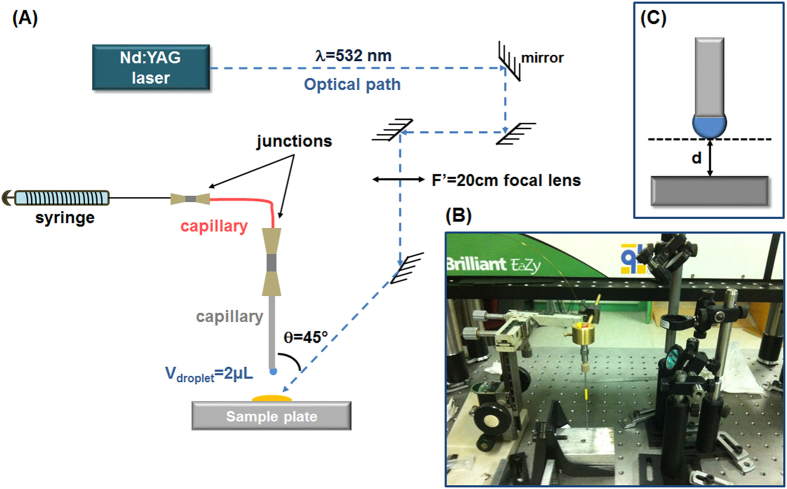
(**A**) Schematic representation and (**B**) photography of the LADC experimental setup. The measurement of the height between the droplet and the sample is schematized in (**C**).

A homemade LADC setup including a 4 ns Nd:YAG Quantel Brilliant Eazy laser operating at 532 nm with a repetition rate of 10 Hz was developed for the experiments (Fig. 9). The laser beam was brought onto the sample surface by coated mirrors and a CaF_2_ convergent lens (f′ = 20 cm). The sample surface was irradiated by the laser beam with an incidence angle of 45°. This angle was required to prevent the interception of the droplet by the laser beam which might lead to its explosion at the laser energy used in the experiments. The surface of averaged ablation spot for 1.4 mJ/pulse at 45° was about 350 × 400 μm which corresponded to a fluence of ≈1 J/cm^2^. A laser power meter (Molectron Coherent Corporate, Santa Clara, CA, USA) was used to measure the laser energy. The solvent delivering system consists of a syringe connected to a stainless steel capillary of 1.5 mm outer diameter and 0.58 mm inner diameter adjusted to deliver droplets of 2 μl at the extremity of the capillary. A solution of 0.1% FA in H_2_O was used as solvent for the LADC experiments. By default, the capillary was at a distance (d) of 1 mm above sample plate. The droplet was positioned above the laser irradiation area to ensure the maximum capture of ablated neutral molecules. After laser ablation and material capture into the droplet, the droplet was manually collected using a micropipette for MALDI-MS analysis.

### Sample preparation for LADC experiments

Optimization of the LADC technique was achieved on standard samples of different biomolecules families including a lipid (PC), a peptide (BK) and a protein (LYZ). PC lipid species (PC 15:0/15:0, M_w_(mono) = 750.531 u.) was suspended in MeOH to obtain a final concentration of 10^−3^ M. Bradykinin peptide (BK, M_w_(mono) = 1060.226 u.), Lysozyme C protein (LYZ, Mw(avg) = 14307 u.), carmine and Congo red dyes were suspended in H_2_O to obtain a final concentration of 10^−3^ M. Cytochrome C was suspended in H_2_O to obtain the same final concentration of 10^−4^ M. The 532 nm absorption coefficients of the samples at their final concentration were measured by visible-near infrared absorption spectroscopy: 0,2 cm^−1^ for pure BK and PC samples, 1 cm^−1^ for carmine, 2.4 cm^−1^ for cytochrome C and 28 cm^−1^ for Congo red. Before LADC, a volume of 2 μl of the standards were deposited onto the sample plate and allowed or not to dry. For liquid samples a homogeneous film is observed while for dried samples, sample evaporation leads to formation of polycrystalline layer of micro-crystals. These micro-crystals are rather homogeneously distributed for certain biomolecules such as BK peptide but highly heterogeneously for PC lipids (most of crystals being found in the outer rim of the deposited droplet). Thicknesses and absorption coefficients were checked by measuring the attenuation of the laser light through the samples deposited on glass slides. We measured that the absorption coefficient of the dry samples remains broadly unchanged compared to the liquids measured under absorption spectroscopy. For BK-Congo red sample according to Beer-Lambert’s law, the obtained values, 16% of absorption for the liquid sample corresponds to a thickness of ~650 μm which is in good agreement with the theoretical estimations derived from the volume deposited and spot area. For the dry sample, the exact thickness cannot be calculated due to the heterogeneity of crystallization but we can estimate by observation that crystals are of size in the range of a tens μm. However, we measured for dry sample 25% of absorption which would give ≈500 μm equivalent “optical” thickness using Beer Lambert’s law. All experiments were achieved in triplicate from different sample spots (1 experiment for each sample spot) or on the same dry deposit in different locations using the pneumatic spray deposit to obtain statistically significant results. For biological tissues analysis, male Wistar rat tissue sections were used for the experiments. Male Wistar rats were used and treated in accordance to the European Communities Council Directive (2010/63/EU) regarding the use of animals in Research, French Law for Animal Protection R214-87 to R214-137 and were approved by the Institutional Animal Care and Use Committee (IACUC) of Lille University. Extraction of the brain was performed as described previously^58^. Briefly, the rats were anesthetized with carbon dioxide and the collected brains were snap-frozen in isopentane (−50 °C). The frozen tissues were then stored at −80 °C until use. 60 μm thickness tissue sections were performed using a cryostat CM1510S (Leica Microsystems, Nanterre, France) and deposited onto the sample plate to perform the LADC experiments. To get significant results, the experiments were achieved on three different points belonging to the same histologic region and on three consecutive tissue sections.

### MALDI-TOF Mass Spectrometry analysis

The droplets containing captured materials were collected and analyzed by MALDI MS. Each captured droplet was directly mixed onto the MALDI plate with 1 μL of matrix solution. Appropriated matrix and standard protocols were used according to the family of analyzed biomolecules i.e. HCCA (10 mg; ACN/TFA 0.1%aq, 7:3, v/v) for peptides, SA (20 mg; ACN/TFA 0.1%aq, 6:4, v/v) for proteins and 2,5-DHB (30 mg; MeOH/TFA 0.1%aq, 1:1, v/v) for lipids. Samples were subsequently analyzed on an Ultraflex II MALDI-TOF/TOF (Bruker Daltonics, GmBH, Bremen, Germany) mass spectrometer equipped with a Smart Beam Laser (modified Nd:YAG, λ = 355 nm) in positive reflectron and linear modes. For each sample, MS spectra were recorded from the accumulation of 2000 laser shots at a repetition rate of 100Hz. Mass spectra were processed with FlexAnalysis 3.2 software (Bruker Daltonics GmBH, Bremen, Germany).

### Data processing for tissues

For tissue sections and thick tissue, peak lists (m/z, intensity) were selected between the mass range m/z 600–1000, using signal-to-noise ratio higher than 3. The spectra of the matrix alone were separately acquired and compared to the data from tissues in order to keep the signals attributed to endogenous tissue compounds only. For these experiments, the sum of the intensity for each significant m/z spectrum per condition was recorded (three points per tissue and three consecutive tissue sections, and three points for thick tissue). To assign the subclass of lipids detected from tissue sections, a lipid extraction using a modified Folch method^59^ was carried out from a rat brain tissue section of 10 μm thickness. Briefly, a volume of 300 μL of CHCl_3_/MeOH (2:1; v/v) was added into a 1.5 mL vial which was then mixed during 1H at room temperature. 50 μL of H_2_O was then added to the solution and the mix was centrifuged 10 minutes at 5000 g (Centrifuge 5415R, Eppendorf). The lower phase was retrieved and dried in a vacuum concentrator (Thermo Scientific, Bremen, Germany). The dried lipid pellet was reconstituted with 100 μL of MeOH/0.1% aq. TFA (7:3; v/v) and 5 μl of the extract was injected by direct infusion on a nanoESI-Q-orbitrap mass spectrometer (Q-Exactive, Thermo Scientific, Bremen, Germany). A full scan MS was acquired during 30 s in the [600–1000] m/z range. The MS/MS spectra were generated in the CID mode using collision energy of 20 eV for all parent masses. The identification of lipid species was performed using LIPIDMAPS database^60^ with a tolerance of +/−0.1 u. in positive mode for the parent mass or by observing some characteristic fragments for lipid species.

### Proteomic application on rat brain tissue section

After the collection of the droplet containing ejected pieces of tissue, a Shotgun strategy was performed. A volume of 4 μl DTT (20 mM in 50 mM AB) was added to the samples and the mix was incubated at 56 °C during 15 min. Thus, the samples were cooled at 4 °C and a volume of 4 μl IAA (100 mM in 50 mM AB) was added and stored at room temperature in the darkness during 15 min. Finally, a volume of 2 μl of trypsin (20 μg/ml in 50 mM AB) was added and the samples were incubated at 37 °C overnight. Samples were desalted on a C-18 ZipTip (Millipore, Saint-Quentin-en-Yvelines, France), dried under vacuum and suspended in ACN/FA0.1%aq (2:98, v/v). The samples were separated by online reversed-phase chromatography using a Thermo Scientific Proxeon Easy-nanoLC system equipped with a Proxeon trap column (100 μm ID x 2cm, Thermo Scientific) and C18 packed tip column (75 μm ID x 15 cm, Acclaim Pepmap 300, Thermo Scientific). Elution was carried out using an increasing gradient of ACN (5% to 35% over 100 min) and a flow rate of 300 nL/min. The chromatography system was coupled to a Thermo Scientific QExactive mass spectrometer programmed to be acquired in a data-dependent mode. The survey scans were acquired in the Orbitrap analyzer operated at a resolving power of 70 000. A mass range of m/z 300–1600 and a target 1E6 were used for the survey scans. The method was set to analyze the top 10 most intense ions from the survey scans to then subject them for ion trap higher-energy collision dissociation (HCD) fragmentation with an isolation window of 1.6 u and normalized collision energy of 30%. Tandem mass spectra were processed with Thermo Scientific Proteome Discoverer software version 1.3. Spectra were searched against UniprotKB/Swiss-Prot filtered with *Rattus norvegicus* taxonomy using the Sequest algorithm (version 1.3.0.339). The search was performed choosing tryspin as the enzyme with one missed cleavage allowed. Precursor mass tolerance was 10 ppm and fragment mass tolerance was 0.5 Da. Cysteine carbamidomethylation was set as fixed modification and methionine oxidation was set as variable modification. The proteins identified, using Sequest with a false-discovery rat of 1%, were then exported to Scaffold version 3.6.2 and Perspectives version 2.0.5 (Proteome software Inc., Portland, Oregon) for label-free quantification based on spectral counts.

## Additional Information

**How to cite this article**: Fatou, B. *et al.* Substrate-Mediated Laser Ablation under Ambient Conditions for Spatially-Resolved Tissue Proteomics. *Sci. Rep.* **5**, 18135; doi: 10.1038/srep18135 (2015).

## Supplementary Material

Supplementary Information

Supplementray data2

Supplementray data3
