# Supplementary material for: Substrate-Mediated Laser Ablation under Ambient Conditions for Spatially-Resolved Tissue Proteomics
Source: Sci Rep. 2015 Dec 17;5:18135. doi: 10.1038/srep18135 (PMC4682183; doi:10.1038/srep18135)
Supplement: Supplementary Information [file srep18135-s1.pdf]

# Substrate Mediated-Laser Ablation under Ambient Conditions for Spatially-Resolved Tissue Proteomics

Benoit Fatou[†, ‡], Maxence Wisztorski[†], Cristian Focsa[‡], Michel Salzet[†], Michael Ziskind[*,‡], Isabelle Fournier[*,†]

[†]Laboratoire Protéomique, Réponse Inflammatoire et Spectrométrie de Masse (PRISM) - INSERM U1192 - Université Lille 1, Bât SN3, 1[er] étage, Cité Scientifique, F-59655 Villeneuve d'Ascq Cedex

[‡]Laboratoire de Physique des Lasers, Atomes et Molécules - CNRS UMR 8523, Bât P5, Université Lille 1, Cité Scientifique, F-59655 Villeneuve d'Ascq Cedex, France

**Corresponding Author**

Prof. Isabelle Fournier.

isabelle.fournier@univ-lille1.fr

Tel: +33 (0)3 20 43 41 94

Fax: +33 (0)3 20 43 40 54

Dr. Michael Ziskind

michael.ziskind@univ-lille1.fr

Tel: +33 (0)3 20 33 63 30

Fax: +33 (0)3 20 33 64 63.

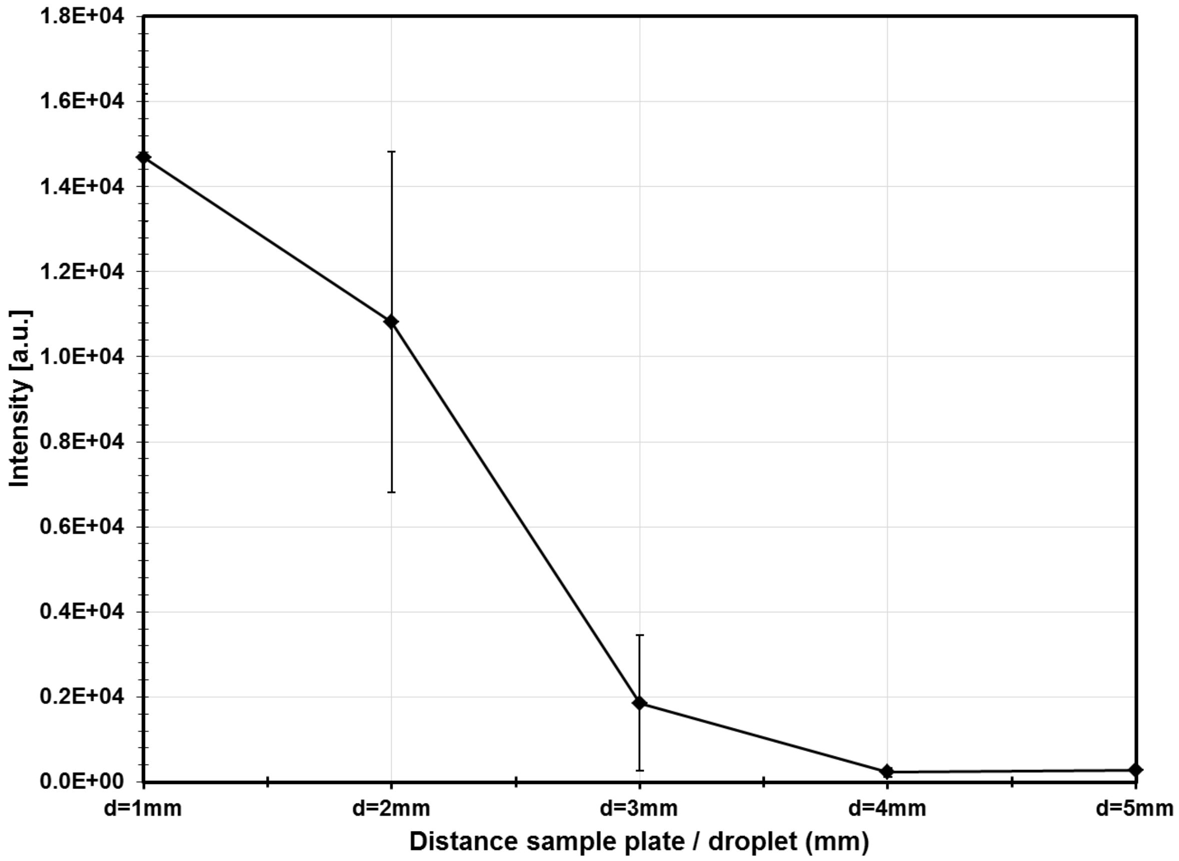


**Figure S-1:** Evolution of the signal intensity as recorded in MALDI MS by analyzing the capture droplet after LADC experiment at 1.4 mJ/pulse LADC laser energy for BK solid sample from stainless steel substrate with the distance d between the sample plate and the capture droplet. Signal intensity corresponds to the averaged intensity of $[M+H]^+$ signals taken over the different measurements.

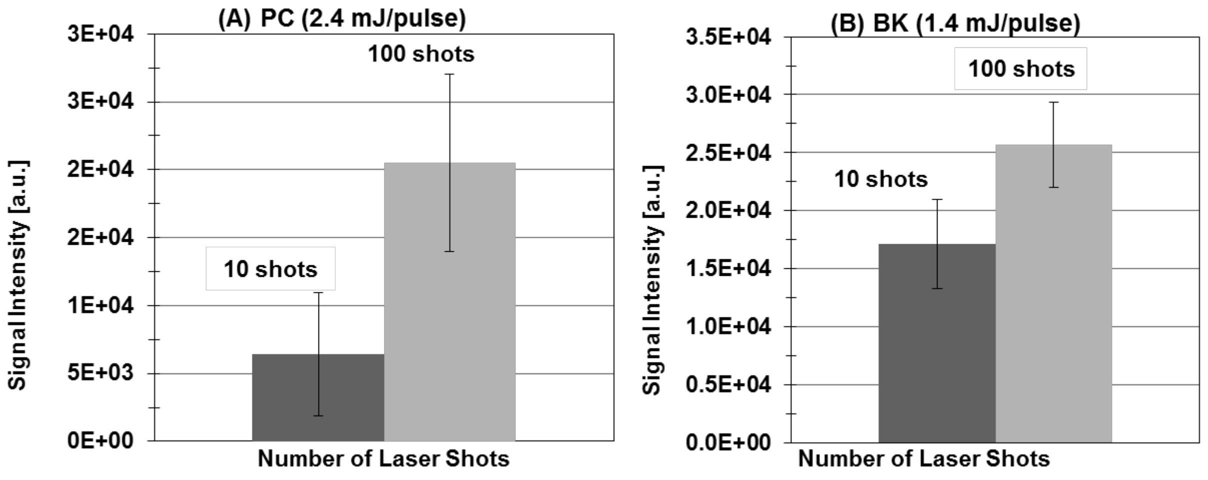


**Figure S-2:** Comparison of the signal intensity as recorded in MALDI MS by analyzing the capture droplet after LADC experiment for liquid PC and solid BK standards respectively at 2.4 mJ/pulse and 1.4 mJ/pulse LADC laser energy with the number of laser shots (10 versus 100 shots). Signal intensity corresponds to the averaged intensity of $[M+H]^+$ signals taken over the different measurements.

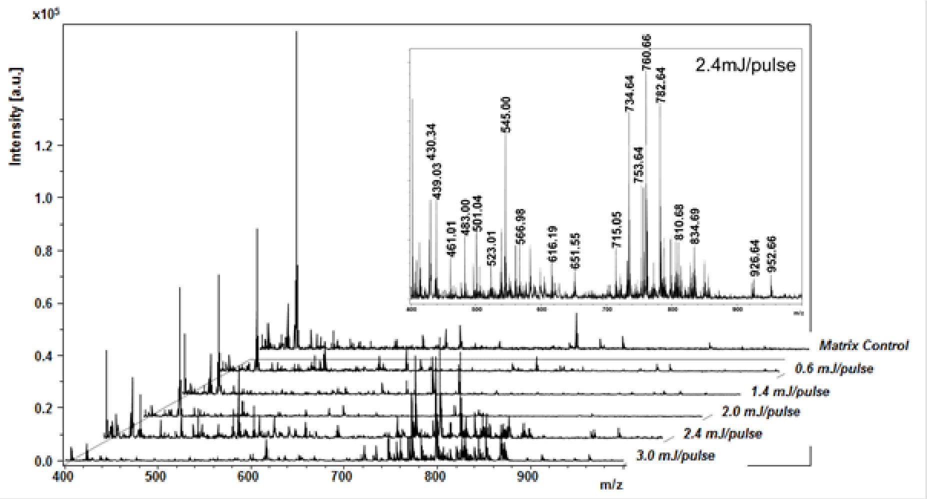


**Figure S-3:** MALDI MS spectra of the capture droplet after LADC experiment from stainless steel substrate on a rat brain tissue section (60 µm thickness) for respectively 0.5, 1.4, 2.0, 2.4 and 3.0 mJ/pulse LADC laser energy/pulse. Top MALDI MS spectrum corresponds to MALDI matrix control recoded in the same conditions. Insert gives a zoom for the MS spectrum at 2.4 mJ/pulse in the [400-1000] m/z range.

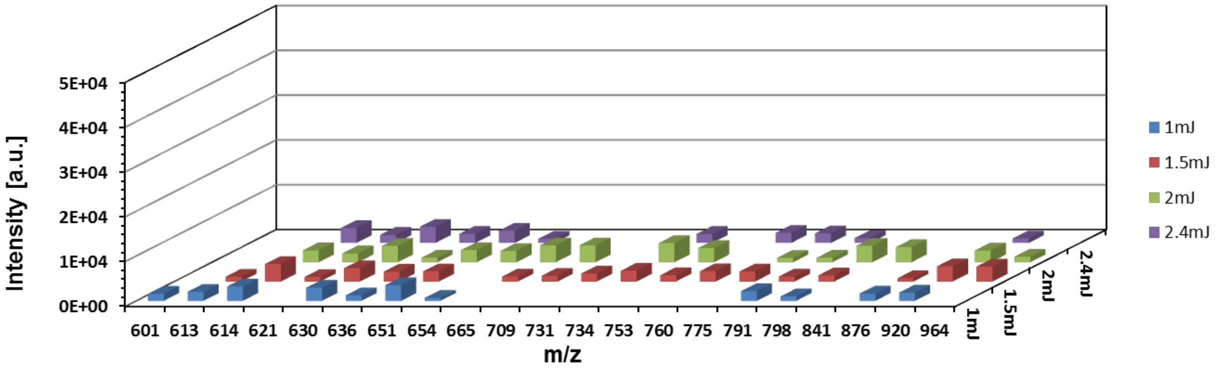


**Figure S-4:** Evolution of the signal profiles (for most significant signals) from stainless steel substrate on a rat brain tissue section (1mm thickness) for 1.0, 1.5, 2.0 and 2.4 mJ/pulse LADC laser energy. Each bar represents the sum of the signal intensity over 3 experiments.

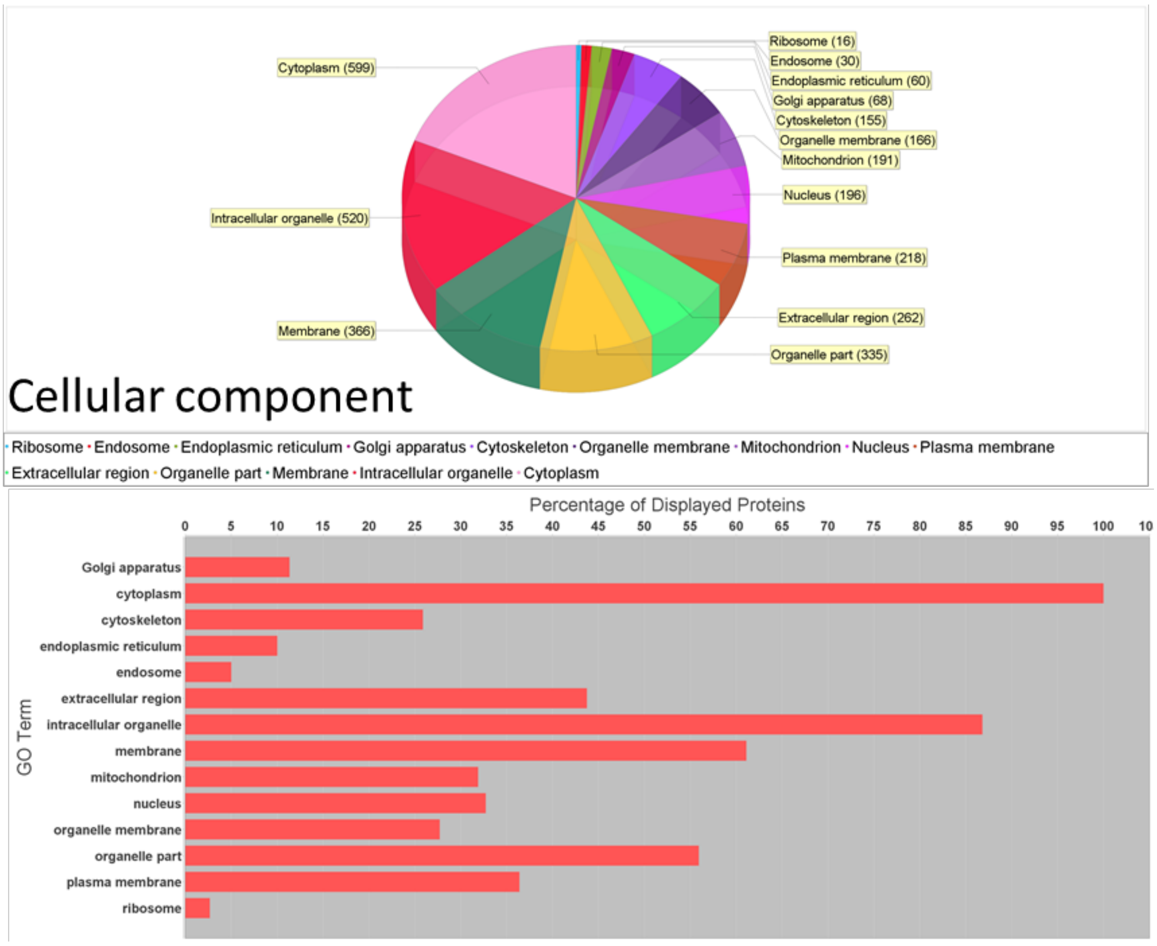


**Figure S-5:** Cellular component distribution (given 2 different representations) of the proteins identified from LADC experiments after Shot-Gun analysis on a rat brain tissue section (60 µm thickness) performed from 19 distinct consecutive irradiation points in a row. For each irradiated point, a single capture droplet is collected and processed for proteomics.

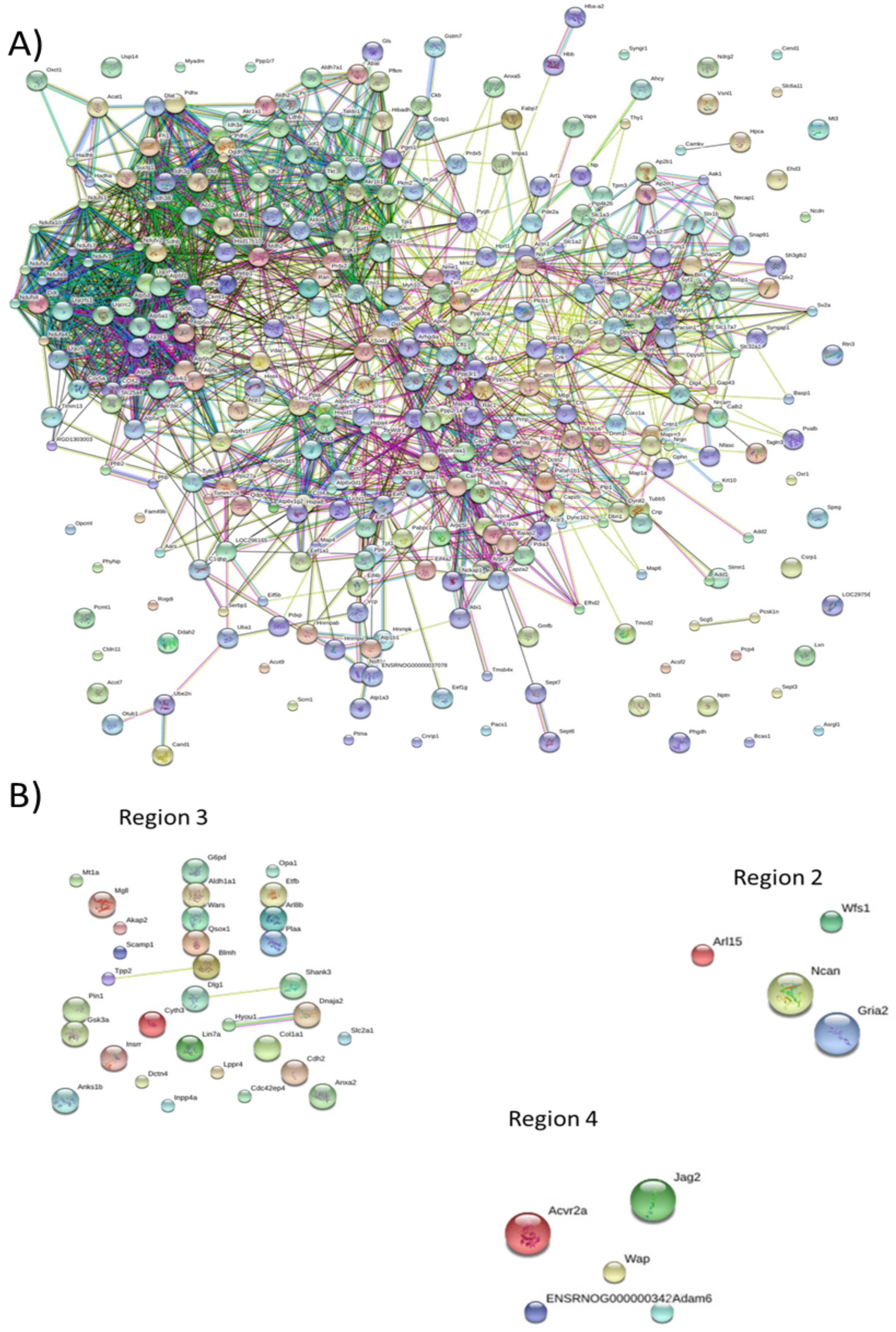


**Figure S-6:** String analysis performed on the proteins identified from LADC experiments after Shot-Gun analysis on a rat brain tissue section (60 µm thickness) performed from 19 distinct consecutive irradiation points in a row. **(A)** Common proteins **(B)** proteins specific to each of the morphological studied region.
